# Impact of BMI on breast screening participation: a data linkage study

**DOI:** 10.1093/eurpub/ckac131.213

**Published:** 2022-10-25

**Authors:** K McBride, S Munasinghe, S Sperendei, A Page

**Affiliations:** 1 School of Medicine, Western Sydney University, Penrith, Australia; 2 Translational Health Research Institute, Western Sydney University, Penrith, Australia

## Abstract

**Background:**

Regular mammographic screening can reduce breast cancer morbidity and mortality. Participation rates are suboptimal in Australia’s fully funded biennial breastscreening program (BreastScreen) for women aged 50-74. Despite obesity being a well-established risk factor for post-menopausal breast cancer, cross sectional data suggests obesity may be a risk factor for non-participation in recommended screening, due to adverse screening experiences. This research aimed to ascertain the link obesity and non-participation by using data linkage of routinely collected data.

**Methods:**

Data for women age eligible for breast screening were linked between the NSW Cancer Registry and the Australian Longitudinal Study of Women’s Health (ALSWH) to create a cohort of women who either participated in screening as recommended or not. Women from the 1946-1951 ALSWH birth cohort were included in the study. These women reported BMI via 8 survey waves. The primary outcome was adherence to breast screening measured by frequency of screening over the follow-up period (1998-2016). Unadjusted risk ratios were calculated using mixed-effects logistic regression for the association between BMI and screening participation.

**Results:**

The study included 2804 linked records of age eligible women (mean age of 52.37[SD 5.47]). 22.8% of the cohort were obese (BMI>30kg/m2). Obesity was significantly associated with non-recommended screening participation (screening within 3 years of last breast screen); odds ratio 1.63 (95% confidence interval 1.32 to 2.00, p < 0.0001).

**Conclusions:**

Obesity has a significantly impact on recommended participation in a nationally provided breast screening program, despite obesity being a risk factor for post- menopausal breast cancer. Optimising participation among higher risk and under-screened women in under utilised breast cancer screening programs is warranted. Development of targeted interventions to increase screening participation among these higher risk women is needed.

**Key messages:**

• Women living with obesity and less likely to participate in recommended breast screening.

• Targeted interventions are needed to optimise participation in breast screening to ensure these higher risk women are not at higher risk of adverse outcomes due to breast cancer.

